# Direct and Indirect Reduction of Cr(VI) by Fermentative Fe(III)-Reducing *Cellulomonas* sp. Strain Cellu-2a

**DOI:** 10.4014/jmb.2107.07038

**Published:** 2021-09-03

**Authors:** Anamika Khanal, Hor-Gil Hur, James K. Fredrickson, Ji-Hoon Lee

**Affiliations:** 1 Department of Bioenvironmental Chemistry, Jeonbuk National University, Jeonju 54896, Republic of Korea; 2 School of Environmental and Earth Science, Gwangju Institute of Science and Technology, Gwangju 61005, Republic of Korea; 3 Pacific Northwest National Laboratory, 902 Battelle Blvd, Richland, WA, 99354, USA; 4 Department of Agricultural Convergence Technology, Jeonbuk National University, Jeonju 54896, Republic of Korea

**Keywords:** *Cellulomonas*, Cr(VI) reduction, Fe(III) reduction, heterogenous reduction

## Abstract

Hexavalent chromium (Cr(VI)) is recognized to be carcinogenic and toxic and registered as a contaminant in many drinking water regulations. It occurs naturally and is also produced by industrial processes. The reduction of Cr(VI) to Cr(III) has been a central topic for chromium remediation since Cr(III) is less toxic and less mobile. In this study, fermentative Fe(III)-reducing bacterial strains (Cellu-2a, Cellu-5a, and Cellu-5b) were isolated from a groundwater sample and were phylogenetically related to species of *Cellulomonas* by 16S rRNA gene analysis. One selected strain, Cellu-2a showed its capacity of reduction of both soluble iron (ferric citrate) and solid iron (hydrous ferric oxide, HFO), as well as aqueous Cr(VI). The strain Cellu-2a was able to reduce 15 μM Cr(VI) directly with glucose or sucrose as a sole carbon source under the anaerobic condition and indirectly with one of the substrates and HFO in the same incubations. The heterogeneous reduction of Cr(VI) by the surface-associated reduced iron from HFO by Cellu-2a likely assisted the Cr(VI) reduction. Fermentative features such as large-scale cell growth may impose advantages on the application of bacterial Cr(VI) reduction over anaerobic respiratory reduction.

## Introduction

Chromium is widely used in diverse industries such as metallurgy, corrosion inhibition, and electroplating and is prevalent in the environment. The risks posed by exposure to the metal ions and compounds are serious, as even, a relatively low level of exposure could cause harms to animals and plants [1-3]. Chromium occurs naturally in rocks, and predominantly in one of two valence states: trivalent Cr which is an essential dietary element, and hexavalent Cr (chromate) which is recognized as a highly toxic and carcinogenic environmental contaminant. Hexavalent chromium contamination in aquatic systems and groundwater occurs also from extensive anthropogenic use of chromate and dichromate in diverse industries. For remediation purposes of the hazardous contaminant, reduction of Hexavalent chromium (Cr(VI)) to Cr(III) has been studied by physicochemical and biological means, and many microorganisms have been shown to reduce Cr(VI) to Cr(III) [4-7].

Microbial reduction of metals has been focused intensively mainly due to its feasibility of immobilizing toxic metals and/or radionuclides including Cr(VI), As(V), U(VI), etc. as well as Fe(III) and Mn(VI) [8-11]. Respiratory reduction of those multivalent metals by *Geobacter* spp., *Shewanella* spp., and sulfate-reducing *Deltaproteobacteria* has been well studied, while less attention has been paid to fermentative microorganisms capable of metal reduction. Recently, *Clostridium acetobutylicum* has been studied as a model system for fermentative iron reduction [12] and fermentative *Cellulomonas* spp. have been reported for the capability of reducing Fe(III) and Cr(VI) as well [5, 6, 13].

Microbial fermentation occurs when electron acceptors are absent under anoxic conditions but with suitable electron donor(s). Despite anaerobic metabolism, fermentation and dissimilatory respiration including metal reduction use different electron transport systems for energy generation. Fermentation processes are considered to occur when external electron acceptors have been depleted. Also, since fermentation is less favorable in terms of energy generation from the organic materials than dissimilatory respiration, fermentation would not proceed to anaerobic respiration utilizing organic carbon in the presence of external electron acceptors [14]. For the expenses of NADH by fermentative bacteria, there should be electron sinks resulting in reduced products, such as pyruvate, in typical fermentation, but other options exist. In dissimilatory metal reduction, electrons derived from oxidation of NADH are transported to the external electron acceptors such as ferric iron and manganese(IV) though a series of cytochromes. In fermentation, those metals might act as electron sinks instead of organic electron acceptors, under high H_2_ accumulation condition [15]. Therefore, although less energetically favorable, metal reduction by the fermentative microorganisms may occur, such as Fe(III) reduction. Here, fermentative Fe(III)-reducing bacteria were sought to examine the capability of direct or indirect reduction of Cr(VI). The results presented herein support a potential use of fermentative bacterial Fe(III) reduction for Cr(VI) detoxification to Cr(III).

## Materials and Methods

### Sample Collection and Enrichment of Iron-Reducing Bacteria

A groundwater sample was collected from a borehole at a depth of 100 m, located in Gunsan, Jeollabuk-do, South Korea (latitude 35°58’13.28’’N and longitude 126°42’56.98’’E). The pH and the electrical conductivity of the water sample were measured using portable probes and a meter (Thermo Scientific, USA). To isolate the Fe(III)-reducing bacteria, enrichment cultures were made by inoculating the groundwater sample using the modified freshwater medium (FW) medium [16, 17] with 10 mM ferric chloride and 10 mM of glucose or sucrose as the sole electron donor.

The modified FW medium containing KH_2_PO_4_ (0.6 g/l), NH_4_Cl (0.3 g/l), MgSO_4_·7H_2_O (0.5 g/l) or MgCl_2_ (0.4 g/l) and CaCl_2_·2H_2_O (0.1 g/l) was prepared at pH of 6.8-6.9 using 22 mM of sterilized sodium bicarbonate buffer. One ml of selenite–tungstate solution [18] was added from the stock solution containing NaOH (0.005 g/l), Na_2_Se_5_H_2_O (0.03 g/l), and Na_2_WO_4_·2H_2_O, and 1 ml of trace element solution [19] was added from the stock solution containing C_6_H_9_NO_6_ (12.8 g/l), FeCl_2_·4H_2_O (2 g/l), CoCl_2_·6H_2_O, MnCl_2_·2H_2_O (100 g/l), ZnCl_2_ (70 g/l), H_3_BO_3_ (6 g/l), NiCl_2_·6H_2_O (24 g/l), CuCl_2_·2H_2_O (g/l), and Na_2_MoO_4_·2H_2_O (36 g/l) to the final volume of 1 liter. One ml of vitamin solution [20] was added from the stock containing sodium phosphate (10 mM), 4–aminobenzoic acid (0.4 mg/l), D(+)-biotin (0.1 mg/l), nicotinic acid (0.10 mg/l), calcium D(+)-panthothenate (0.10 mg/l), pyridoxine dihydrochloride (0.15 mg/l), thiamine (0.10 mg/l), and vitamin B12 (0.5 mg/l). The FW medium was purged for 30 min using N_2_:CO_2_ (90:10) gas. The stock solutions of ferric chloride (FeCl_3_) and sodium nitrate (NaNO_3_) were prepared separately and N_2_ gas was purged to create strict anaerobic conditions.

Serum bottles containing 50 ml of modified FW media were tightly closed with butyl rubber stoppers and aluminum seals. Ferrous chloride and sodium nitrate were added to the serum bottle, resulting in a final concentration of 10 mM and 5 mM, respectively. One percent of the groundwater sample was added and incubated in darkness at 28°C for 2 weeks. After the enrichment incubations, 1% of the culture was transferred to fresh media, and then 100 μl of the culture from the serum bottle was spread on the FW agar plate media prepared anaerobically with 10 mM of FeCl_3_ and 5 mM of NaNO_3_. Plates were kept in the anaerobic jars (Merck KGaA, Germany) with the anaerobe atmosphere generation bags in darkness at 28°C for 2 weeks, while the colonies were observed. The observed individual colonies on the plates were transferred on fresh agar plates up to 4 times in anaerobic conditions and the colonies were purified. The purity of the isolates was checked with gram staining by light microscopy.

### Phylogenetic Analysis on the Bacterial Isolates

The isolated colonies were cultured in R2A broth aerobically at 28°C for two days and genomic DNAs were extracted using an InClone genomic plus DNA preparation kit (Inclone biotech, Korea). DNA quantity was measured by fluorometry (Qubit 3.0 Fluorometer, Invitrogen, USA). A total of 50 μl of the reaction mixture was prepared for the PCR reactions on 16S rRNA gene, where 1-2 μl of the diluted genomic DNAs (1-5 ng/μl) and 1 μl of each primer (27F, 5'-AGA GTT TGA TCM TGG CTC AG-3'; 1492R, 5'-GGT TAC CTT GTT ACG ACT T-3') at the final 0.2 μM were added. The thermal cycles were performed with an initial denaturation at 95°C for 2 min, 30 cycles of denaturation at 94°C for 30 sec, annealing at 50°C for 30 sec, and an extension at 72°C for 1 min 30 sec. The final extension was performed at 72°C for 5 min. The quality and quantity of the purified DNA was checked respectively using gel electrophoresis and a fluorometer.

The purified PCR products of 16S rRNA gene with approximately 1500 bp-long were sequenced at GenoTech (Korea), and the results of forward and the reverse sequences were assembled. The assembled and manually corrected sequences were compared to known 16S rRNA gene sequences from the NCBI GenBank using BLAST search [21]. Phylogenetic trees were constructed with the aligned sequences retrieved from SILVA Release 138.1 Ref NR 99 database [22] in ARB 6.0.6 [23]. The evolutionary history was inferred using the neighbor-joining method [24]. The nucleotide sequences were deposited to GenBank with the accession number of MT012157-MT012159.

### Influence of Carbon Source on Iron Reduction

Biochemical characterization of the isolated bacterial strains (Cellu-2a, Cellu-5a, and Cellu-5b) was performed using API 50 CH and API 50 CHB/E media (bioMerieux, France). The change in color of the strips was observed after 24 h and 48 h, where the substrates are utilized by the microorganisms from red to yellow.

Optimization of carbon source for iron reduction was conducted. Prior to the iron reduction experiment, poorly crystalline hydrous ferric oxide (HFO) was prepared by titrating 150 ml of 0.1 M Fe(NO_3_)_3_ solution using approximately 50 ml of 1.0 N NaOH up to pH 8. The precipitated material was washed with distilled water 3 times by centrifugation/resuspension, at which point 100 ml of distilled water was added and then purged with 100% N_2_. The concentration of ferrous iron was measured by the ferrozine method [25] at 562 nm. From the incubations using ferric-citrate or HFO, Fe(II) was measured after 0.5 N HCl extraction, and the total Fe was measured by reducing whole Fe to Fe(II) using NH_2_OH·HCl, which was used to calculate Fe(III) concentration.

For the iron reduction experiment, 50 ml modified FW media were supplemented with four different carbon sources of lactates, glucose, sucrose, or acetate at 10 mM in 100 ml serum bottles. To test for soluble iron reduction, a final concentration of 2 mM ferric-citrate was added, while to test for solid iron reduction, a final concentration of 2 mM HFO was used. Serum bottles were sealed with butyl rubber stopper, and N_2_:CO_2_ (90:10) gas was purged in the media and head space to develop anaerobic condition. The isolated strain Cellu-2a was then added to each serum bottle at the final cell concentration of 2 × 10^6^ cells/ml by manipulating optical density at 600 nm. Abiotic control incubations were performed using the same conditions, in both (soluble and solid iron) cases without inoculation of any microorganism.

### Chromium Reduction

Cr(VI) as potassium chromate (K_2_CrO_4_), reduction was assessed by the isolated strains. Direct and indirect reduction experiments were performed with or without addition of HFO in the modified FW media. For the direct reduction experiment 15 μM Cr(VI) was added to the modified FW media, whereas for the indirect reduction experiment 15 μM Cr(VI) and 2 mM HFO were added. Ten mM of glucose or sucrose was added as a carbon source. The retrieved subsamples were filtered by 0.45 μm syringe filters and aqueous Cr(VI) was measured by USEPA 1,5-diphenylcarbohydrazide method (Method 8023, HACH, USA) with absorbance at 540 nm.

### Transmission Electron Microscopic (TEM) and Energy Dispersive X-Ray Spectroscopy (EDS)

For the TEM analysis of the cells and the associated particles, the samples were removed from the serum bottles using aseptic syringes then diluted in sterile anaerobic water and dried on carbon-coated copper grids. Images of the whole mounts were taken at 100 kV using a Bio-TEM (H-7650, Hitachi, Japan). Field emission TEM with EDS (JEM 2100F, Jeol, Japan) was used to observe the particles and identify the elemental composition of materials.

## Results

### Bacterial Isolation from the Groundwater

The collected groundwater showed pH of 8.76 and electrical conductivity of 13.18 ds/m. Concentrations of nitrate and ammonium were determined to be 0.59 μM and 0.149 mM, respectively. Through a series of enrichment and agar streak-plate transfers, several colonies capable of Fe(III) reduction were isolated from the groundwater. The isolated bacterial strains of Cellu-2a, Cellu-5a, and Cellu-5b were capable of anaerobic fermentative growth in the modified FW media, supplemented with glucose as a sole carbon source. The isolated strains were able to grow in several tested media, including R2A, lysogeny broth (LB), nutrient broth (NB), and tryptic soy broth (TSB) media at 28°C in both anaerobic and aerobic conditions.

### Biochemical Test and Phylogenetic Analysis

The isolated strains (Cellu-2a, Cellu-5a, and Cellu-5b) showed similar substrate utilization patterns. Strain Cellu-2a utilized L–arabinose, D-xylose, D-glucose, D-fructose, D-mannose, D-mannitol, N-acetylglucosamine, esculin ferric citrate, salicin, D-saccharose, D-cellobiose, and amidon (starch) after 24 h, and utilized D-galactose and D-trehalose after 48 h. Strain Cellu-5a utilized L–arabinose, D-xylose, D-glucose, D-fructose, D-mannose, D-mannitol, esculin ferric citrate, D-saccharose, D-cellobiose, amidon (starch), and glycogen after 24 h, and utilized D-galactose, salicin, N-acetylglucosamine, and D-trehalose after 48 h. Strain Cellu-5b utilized L–arabinose, D-xylose, D-glucose, D-fructose, D-mannitol, esculin ferric citrate, D-saccharose, D-cellobiose, amidon, and glycogen after 24 h, and utilized D-mannose, D-galactose, salicin, N-acetylglucosamine, D-maltose, and D-trehalose after 48 h.

**Fig. 1:**
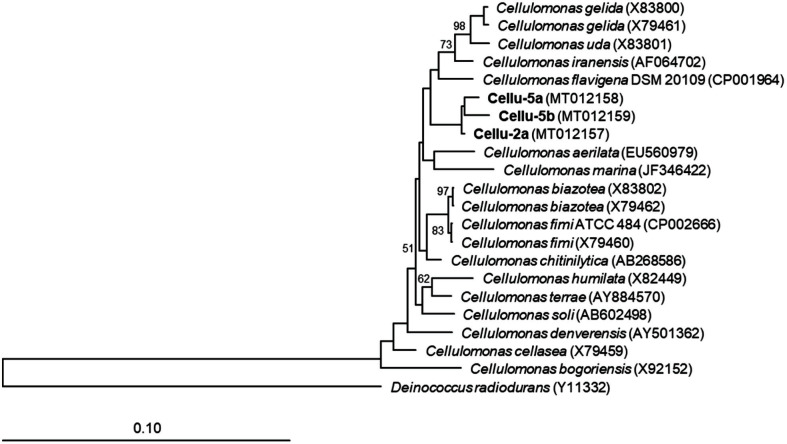
Neighbor-joining phylogenetic tree of 16S rRNA gene sequences of the isolated strains, Cellu-2a, Cellu-5a, and Cellu-5b.

Amplification of 16S rRNA genes of the isolated strains using the 27F and 1492R universal primers revealed approximately 1.4 kb DNA band in 1% agarose gel. The sequences showed similarities to *Cellulomonas* species (Fig. 1).

### Reduction of Ferric Iron

Among the isolated strains of Cellu-2a, Cellu-5a, and Cellu-5b, strain Cellu-2a was selected for further study due to its ability to use a wider range of carbon sources fast. By preliminary tests, the strain Cellu-2a was able to reduce ferric-citrate with glucose or sucrose as the carbon source among the tested four different organic carbon substrates of glucose, sucrose, acetate, and lactate.

**Fig. 2:**
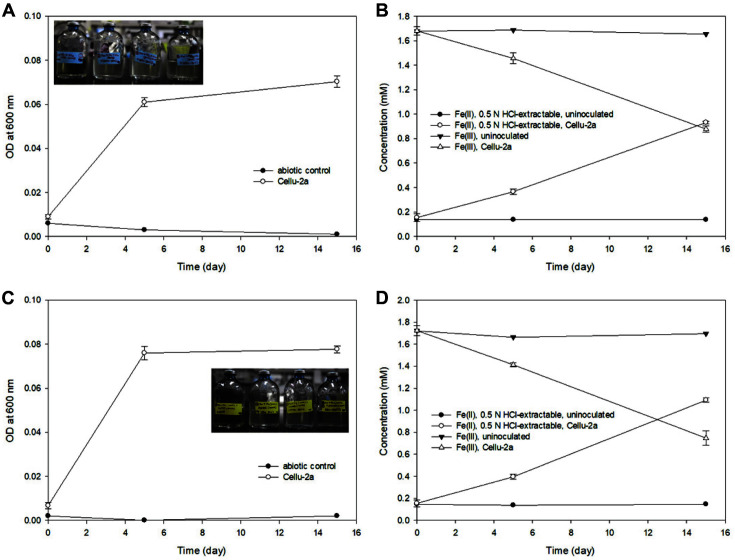
Cell growth curves and Fe concentrations during 2 mM ferric-citrate reduction by Cellu-2a using glucose (A, B) or sucrose (C, D) as a carbon source. The subset photos taken at day 15.

**Fig. 3:**
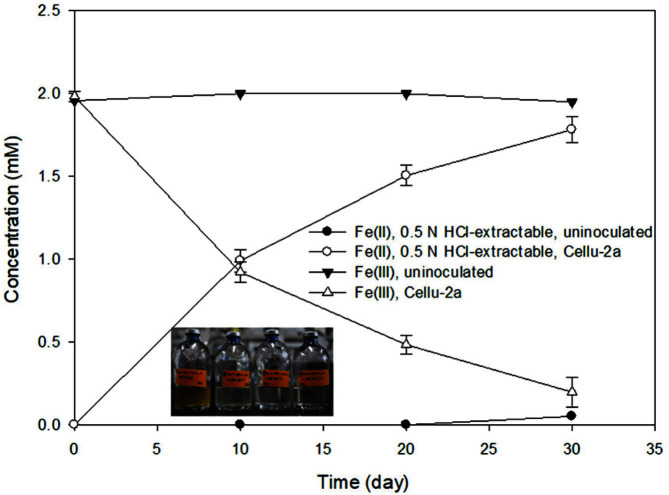
Concentrations of Fe during 2 mM HFO reduction by strain Cellu-2a, incubated with 10 mM sucrose as a sole carbon source. The subset photo taken at day 30.

Strain Cellu-2a reduced ferric-citrate (initially approximately 1.8 mM) with glucose as a sole carbon source (Fig. 2A), generating approximately 0.9 mM Fe(II)_(aq)_ in 15 days (Fig. 2B). When sucrose was used as a carbon source (Fig. 2C), approximately 1.1 mM ferric iron was reduced in 15 days (Fig. 2D). Bacterial growth was observed in the ferric-citrate reduction, and no significant reduction of Fe(III) was observed in the abiotic/uninoculated controls with glucose or sucrose. Since the Fe(III) reduction rates using glucose and sucrose were not significantly different, sucrose was selected as a carbon source for further experiments with solid iron. With the solid ferric iron mineral of HFO, nearly 92% Fe(III) of 2 mM HFO was reduced in the incubations inoculated with the strain Cellu-2a in 30 days (Fig. 3).

### Direct and Indirect Reduction of Chromium

**Fig. 4:**
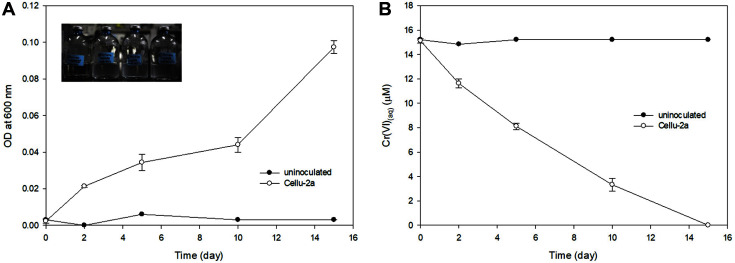
Incubation for direct Cr(VI) reduction by Cellu-2a with 10 mM sucrose. **A**. Optical density with or without Cellu-2a; **B**. Cr(VI) concentrations with or without Cellu-2a. The subset photo taken at day 15.

**Fig. 5:**
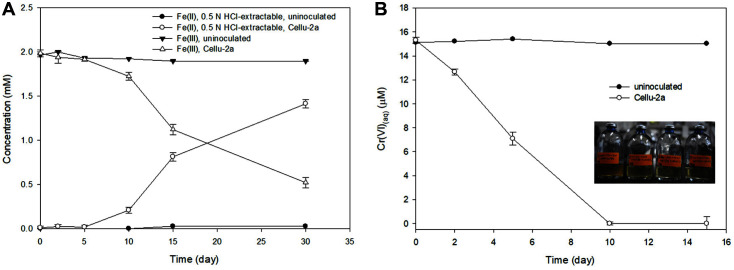
Incubation for indirect Cr(VI) reduction by Cellu-2a in the presence of 2 mM HFO and 10 mM sucrose. **A**. Concentrations of Fe(II) and Fe(III) with or without Cellu-2a; **B**. Cr(VI) concentrations with or without Cellu-2a. The subset photo taken at day 15.

Cr(VI) reduction was observed in the incubation of the strain Cellu-2a in modified FW medium supplemented with 10 mM sucrose and 15 μM Cr(VI). In 15 days nearly 15 μM Cr(VI) was reduced and cell growth was observed (Fig. 4). From the separated bacterial incubations in the FW medium with 10 mM sucrose, 15 μM Cr(VI), and 2 mM HFO, the reduction of Cr(VI) was observed, while none was observed in the abiotic control (Fig. 5). With gradual reduction, Cr(VI) was reduced nearly completely in 10 days. The Fe(III) reduction was not significant up to day 5 but increased by day 10 (Fig. 5A). This result indicated that indirect Cr(VI) reduction via reduced iron was faster than direct Cr(VI) reduction by Cellu-2a cells which showed a gradual decrease up to 15 days (Fig. 4B).

### TEM and EDX Analyses

**Fig. 6:**
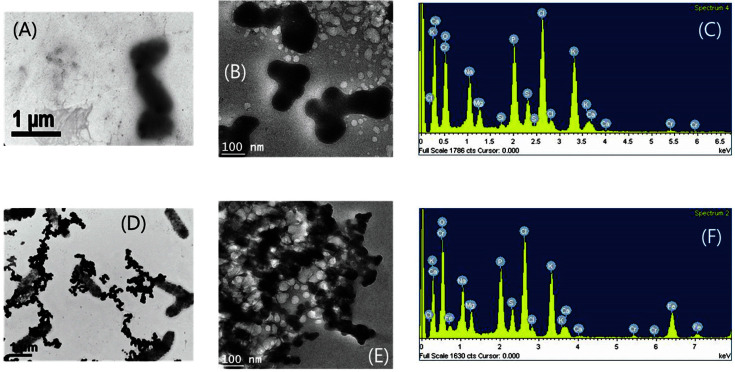
TEM images and EDS spectra of bacterial incubations for direct (A, B, and C) and indirect (D, E, and F) reduction Cr(VI).

TEM and EDX analyses were performed on the precipitated samples, collected at the end of the experiments, 20 days of direct Cr(VI) reduction and 40 days of indirect Cr(VI) reduction. As aqueous Cr(VI) has been known to be reduced to Cr(III) [4], the dark precipitates observed from the direct Cr(VI) reduction incubations were speculated to be insoluble Cr(III) particles, such as Cr(III) hydroxides (Figs. 6A and 6B), where Cr(III) could be coordinated possibly with the functional groups of glycolipids on the bacterial cell surface [4]. Chromium particles were observed outside the cells (Fig. 6B). EDS analysis revealed chromium peaks in the spectra of the precipitates from the direct chromium reduction experiment (Fig. 6C). In comparison, both iron and chromium peaks were observed in the spectra of the precipitates collected from the indirect chromium reduction experiment (Fig. 6F). In the indirect Cr(VI) reduction incubations with the HFO, reduced iron minerals were very distinctive (Figs. 6D and 6E), compared to the presumable Cr(III) hydroxide particles in the direct incubations (Fig. 6B).

## Discussion

In this study, the three isolated strains of Cellu-2a, Cellu-5a, and Cellu-5b with more than 99 % rRNA sequence similarities with *Cellulomonas* species. They exhibited fermentative growth using glucose or sucrose as a sole carbon source, consistent with previous studies reporting that some of the *Cellulomonas* were able to degrade cellulose under both aerobic and anaerobic conditions and also fermentatively [26, 27]. In addition, the isolated strains were able to reduce both soluble (*i.e.*, ferric-citrate) and insoluble (*i.e.*, HFO) Fe(III) in fermentative condition.

Ferric iron reduction during fermentation has been previously shown [15, 28, 29], with Fe(III) being speculated to act as an electron sink during the fermentation. A Gram-positive, sulfate-reducing bacterium, *Desulfotomaculum* reducens MI-1 was able to reduce Fe(III) during pyruvate fermentation [15], which was proposed not to support to energy conservation. However, it was suggested that electron transfer to Fe(III) could facilitate pyruvate oxidation presumably to acetate, even under high H_2_ accumulation condition precluding further reduction of protons.

Bacteria from several different genera including *Bacillus* and *Actinobacteria*, are known to perform iron reduction under anaerobic and pH-neutral conditions [30, 31]. Therefore, it can be expected that *Cellulomonas* belonging to *Actinobacteria* might show the growth and/or reduction of iron in conditions where different carbon sources such as glucose, sucrose, lactate, or acetate are used. The isolated strains of Cellu-2a, Cellu-5a, and Cellu-5b showed growth in anaerobic conditions when glucose was used as a carbon source and did not grow using lactate or acetate as an electron donor. A similar result was suggested by Gerlach *et al*. [13], reporting that sucrose and xylose supported anaerobic Fe(III) reduction by a strain of *Cellulomonas*, but lactate and other short-chain organic compounds could only support aerobic growth, not Fe(III) reduction. Probably those short-chain organic compounds are poor substrates for fermentations, but rather normally end products of fermentations.

Although toxic effect of Cr(VI) on bacterial growth has been reported in microorganisms such as *Shewanella oneidensis* MR-1 [32], the isolated strain Cellu-2a was able to grow in the presence of 15 μM Cr(VI) and reduce the soluble Cr(VI). In addition, chromium was reduced faster with HFO added to the Cellu-2a cultures, than the cultures without addition of HFO. It has been known that Fe(II)-bearing solid phases can act as potent reductants, providing reactive surface sites that can sorb aqueous oxidants and couple the electron transfer between them [33]. So, in this study, Fe(II)-bearing phases produced during the reduction of HFO by Cell-2a and Fe(II) associated with Fe(III) solids might have acted as reductant for aqueous Cr(VI) reduction to Cr(III). This heterogenous reduction reaction would have accelerated the rate of Cr(VI) reduction rather than the direct reduction of Cr(VI) by the cells [34]. Looking at the ferrous iron concentrations generated from the bacterial HFO reduction, the gradual increase rate was diminished in the presence of Cr(VI) (Fig. 5 A) compared to Fe(II) increase rate without Cr(VI) (Fig. 3). This might suggest that heterogenous reduction of Cr(VI) oxidized surface-bound Fe(II). While a great deal of research has focused on the process of reducing heavy metals through respiration, less attention has been paid to iron-reducing, fermentative bacteria. The results of this study might be useful to developing cost-effective and sustainable remediation methods for heavy metal contamination.
